# Comparison of the prevalence, severity, and risk factors for hepatic steatosis in HIV-infected and uninfected people

**DOI:** 10.1186/s12876-019-0969-1

**Published:** 2019-04-15

**Authors:** Jessie Torgersen, Kaku So-Armah, Matthew S. Freiberg, Matthew B. Goetz, Matthew J. Budoff, Joseph K. Lim, Tamar Taddei, Adeel A. Butt, Maria C. Rodriguez-Barradas, Amy C. Justice, Jay R. Kostman, Vincent Lo Re

**Affiliations:** 1 Department of Medicine, Perelman School of Medicine, University of Pennsylvania, 3910 Powelton Ave 4nd Floor, Ste. 411F, Philadelphia, PA 19104 USA; 2 Department of Medicine, Boston University School of Medicine, Boston, MA USA; 3 Department of Medicine, Vanderbilt University School of Medicine, Nashville, TN USA; 4 Department of Medicine, David Geffen School of Medicine at UCLA and VA Greater Los Angeles Healthcare System, Los Angeles, CA USA; 5 Department of Medicine, David Geffen School of Medicine at UCLA, Los Angeles, CA USA; 6 Department of Medicine, Yale University School of Medicine, New Haven, CT USA; 7 Department of Medicine, Yale University School of Medicine, New Haven, CT, USA, and VA Connecticut Healthcare, West Haven, CT USA; 8 Department of Medicine, Weill Cornell Medical College, Ar-Rayyan, Qatar; 9 Department of Medicine, Michael E. DeBakey VA Medical Center, Houston, TX USA; 10 John Bell Health Center, Philadelphia FIGHT, Philadelphia, PA USA; 11 Department of Medicine and Biostatistics and Epidemiology, Perelman School of Medicine, University of Pennsylvania, Philadelphia, PA USA

**Keywords:** Hepatic steatosis, HIV, Liver fibrosis

## Abstract

**Background:**

Hepatic steatosis is prevalent in Western countries, but few studies have evaluated whether the frequency and severity of steatosis are greater in the setting of HIV infection. We compared the prevalence and severity of hepatic steatosis between HIV-infected (HIV+) and uninfected persons and identified factors associated with greater steatosis severity within both groups.

**Methods:**

We performed a cross-sectional study among participants without cardiovascular disease who participated in a substudy of the Veterans Aging Cohort Study. Hepatic steatosis was defined by noncontrast computed tomography (CT) liver-to-spleen (L/S) attenuation ratio < 1.0. Multivariable linear regression was used to: 1) evaluate the association between HIV infection and severity of hepatic steatosis, as measured by absolute liver attenuation, and 2) identify factors associated with greater severity of steatosis, by HIV status.

**Results:**

Among 268 participants (median age, 55 years; 99% male; 79% black; 23% obese; 64% HIV+ [91% on antiretroviral therapy]), the overall prevalence of steatosis was 7.8% and was similar between HIV+ and uninfected individuals (13 [7.6%] versus 8 [8.2%], respectively; *p* = 0.85). Participants with HIV, the majority of whom received antiretroviral therapy, had a higher mean absolute liver attenuation (mean difference, 5.68 Hounsfield units; *p* < 0.001), correlating with lesser hepatic steatosis severity, compared to uninfected participants. After adjusting for covariates, only advanced hepatic fibrosis was associated with greater severity of steatosis in HIV+ persons (*p* = 0.03) and uninfected individuals (*p* < 0.001).

**Conclusions:**

In this sample of participants without cardiovascular disease, the prevalence of hepatic steatosis by noncontrast abdominal CT was not different by HIV status. Increasing severity of steatosis was independently associated with advanced hepatic fibrosis in both groups.

**Electronic supplementary material:**

The online version of this article (10.1186/s12876-019-0969-1) contains supplementary material, which is available to authorized users.

## Background

Liver disease is a leading cause of morbidity and mortality in HIV-infected (HIV+) individuals [1, 2]. Chronic viral hepatitis accounts for the majority of this disease burden, but hepatic steatosis has emerged as a potential contributor to liver fibrosis progression in this population [3, 4]. Existing studies provide conflicting results on the prevalence of hepatic steatosis in HIV infection, and it remains unclear if hepatic steatosis is more common or more severe among HIV+ than uninfected persons. Studies examining hepatic steatosis in HIV have generally been limited to persons with hepatitis C virus (HCV) coinfection, did not evaluate the severity of steatosis by HIV status, and examined only a limited number of risk factors [5–15]. Consequently, additional investigations are needed to assess the prevalence, severity, and determinants of hepatic steatosis in HIV+ persons and compare these to uninfected persons to understand how this disease process may differ by HIV status.

We compared the prevalence and severity of hepatic steatosis, as determined by noncontrast abdominal computed tomography (CT), between HIV+ and uninfected persons within a subcohort of the Veterans Aging Cohort Study (VACS) and evaluated risk factors for greater severity of steatosis within both groups.

## Methods

### Study design and setting

We conducted a cross-sectional study among participants at three sites (Los Angeles, CA; Baltimore, MD; Pittsburgh, PA) in the VACS, an observational prospective cohort study that follows HIV+ and age-, sex-, race/ethnicity-, and site-matched HIV-uninfected Veterans from eight Veterans Health Administration (VA) facilities across the United States [16]. Participants completed standardized questionnaires at VACS enrollment and annually on alcohol/substance use [16, 17]. Demographic and clinical data were available within the VA’s Centralized Patient Record System.

### Study participants

Between 2008 and 2011, a subset of this cohort (171 HIV+ and 97 uninfected) at three sites (Los Angeles, CA; Baltimore, MD; Pittsburgh, PA) consented to undergo noncontrast CT imaging of the chest and abdomen, including scans of the liver and spleen, as a means to evaluate the prevalence and determinants of subclinical cardiovascular disease by HIV status. Patients at the three sites were eligible if they were free of cardiovascular disease (i.e., no prior diagnosis of myocardial infarction, congestive heart failure, or ischemic stroke, and no history of percutaneous coronary intervention or coronary artery bypass graft as assessed by self-report and review of medical records). All participants who were free of cardiovascular disease and who consented to undergo noncontrast abdominal CT imaging were included in this secondary analysis.

### Noncontrast abdominal CT measurements

A 2.5–3.0 mm slice image was obtained through the mid-liver and spleen by noncontrast abdominal CT. Attenuation values, reported in Hounsfield Units (HU), were determined within two regions of the right hepatic lobe and one region of the spleen. Absolute liver attenuation was calculated as the mean value of the two right hepatic lobe measurements [18]. Because absolute liver attenuation/absolute spleen attenuation ratio (L/S) < 1.0 has 94% specificity for diagnosis of hepatic steatosis, defined as ≥5% hepatic triglyceride content by pathology, we chose L/S < 1.0 to define the presence of hepatic steatosis [19]. The severity of hepatic steatosis was defined by absolute liver attenuation (in HU), which has a linear, inverse association with the severity of steatosis by pathology (i.e., higher HU indicates less steatosis) [20]. Absolute liver attenuation < 40 HU accurately identifies ≥30% hepatic steatosis; values ≤25 HU indicate > 50% steatosis. [20] Visceral adipose tissue (VAT) and subcutaneous adipose tissue (SAT) area were also obtained from abdominal CT images using standardized techniques [21].

### Data collection

Demographic, clinical, and laboratory data obtained closest, but prior to the date of the CT scan were used. They included age, sex, race/ethnicity, obesity (body mass index [BMI] ≥30 kg/m^2^), alcohol use (determined by the Alcohol Use Disorders Identification Test-Consumption questionnaire and alcohol dependence/abuse diagnoses and classified as not current, non-hazardous, hazardous, or abuse/dependence), HIV infection (positive HIV antibody or RNA), diabetes mellitus (defined by International Classification of Diseases, Ninth Revision, diagnosis, anti-diabetic treatment, or random glucose > 200 mg/dL), hypertension (blood pressure ≥ 140/90 mmHg or use of antihypertensive medication), and HCV infection (positive HCV antibody or RNA) [22, 23]. Antiretroviral therapy (ART) was defined as use of at least three antiretrovirals from two different classes within 180 days prior to the CT scan [24].

Laboratory variables included current and nadir CD4 cell count, alanine aminotransferase (ALT), aspartate aminotransferase (AST), platelet count, total cholesterol, high-density lipoprotein, and triglyceride levels. The Fibrosis-4 Index for Liver Fibrosis (FIB-4), a non-invasive measure of hepatic fibrosis, was calculated, with FIB-4 scores < 1.45 identifying no/minimal hepatic fibrosis, and scores > 3.25 indicating advanced fibrosis/cirrhosis [25].

### Statistical analysis

The prevalence of hepatic steatosis (L/S ratio < 1.0) was determined by HIV status. Multivariable linear regression was used to evaluate the association between HIV infection and severity of hepatic steatosis, as measured by absolute liver attenuation (in HU), after adjustment for BMI, alcohol use category, diabetes, hypertension, HCV status, triglycerides, and FIB-4. Multivariable linear regression was then performed separately among HIV+ and uninfected participants to identify determinants of increasing severity of steatosis within each group. Since BMI may be an insensitive surrogate for adiposity, we calculated the correlation (Spearman coefficient) between BMI and both VAT and SAT. We assessed the stability of parameter estimates in multivariable models utilizing VAT and SAT in place of BMI [26]. As risk factors for hepatic steatosis may be different in obese and non-obese individuals, we explored associations between covariates and severity of hepatic steatosis stratified by obese BMI and HIV status.

We implemented multiple imputation to address the potential bias of unmeasured AST, ALT, or platelet counts to derive FIB-4, by means of 20 imputations using all variables in Table 1 [27]. This generated 20 complete imputed datasets on which regression analyses were conducted. Results across the datasets were combined to arrive at confidence intervals (CI) that accounted for within- and across-dataset variances. Since regression parameter estimates from models with imputed FIB-4 results were similar to those from models excluding participants with missing FIB-4, the results of models with imputed FIB-4 are presented. All analyses were performed using Stata 13.1 (College Station, TX).

**Table 1:** Characteristics of the study participants, by HIV status

Characteristic	HIV+ (*n* = 171)	HIV-(*n* = 97)	*P*
Median age, years (IQR)	54.5 (50.4, 59.5)	55.5 (51.6, 59.9)	0.33
Male sex, n (%)	170 (99%)	97 (100%)	0.45
Race/ethnicity, n (%)			0.40
White	26 (15%)	10 (10%)	
Black/African American	131 (77%)	82 (84%)	
Hispanic	11 (6%)	3 (3%)	
Other	3 (2%)	2 (2%)	
Body mass index, n (%)			0.007
< 18.5 kg/m^2^	4 (2%)	0	
18.5–24.9 kg/m^2^	70 (41%)	30 (31%)	
25.0–29.9 kg/m^2^	68 (40%)	34 (35%)	
≥ 30.0 kg/m^2^	29 (14%)	33 (34%)	
Median SAT area, cm^2^ (IQR)	183.4 (107.4, 270.5)	251.2 (184.2, 348.0)	< 0.001
Median VAT area, cm^2^ (IQR)	95.3 (48.7, 142.7)	106.4 (58.4, 148.5)	0.39
Diabetes mellitus^a^, n (%)	33 (19%)	19 (20%)	0.95
Hypertension^b^, n (%)	140 (82%)	80 (83%)	0.77
Alcohol consumption^c^, n (%)			0.28
Not current	59 (34%)	29 (30%)	
Non-hazardous	33 (19%)	12 (12%)	
Hazardous/at risk	13 (8%)	9 (9%)	
Dependence/abuse	66 (39%)	47 (48%)	
Current ART use, n (%)^d^	156 (91%)	–	–
Median years on ART (IQR)	6.3 (3.8–9.6)	–	–
Median HIV RNA, copies/mm^3^ (IQR)	48 (40, 72)	–	–
HIV RNA < 75 copies/mm^3^, n (%)	141 (82%)	–	–
Median nadir CD4 count, cells/mm^3^ (IQR)	196.5 (79, 320)	–	–
Median current CD4 cell count, cells/mm^3^ (IQR)	462 (300, 673)	–	–
Current CD4 cell count category, n (%)		–	–
< 200 cells/mm^3^	19 (11%)		
200–350 cells/mm^3^	39 (23%)		
350–500 cells/mm^3^	35 (20%)		
> 500 cells/mm^3^	77 (45%)		
Missing	1 (1%)		
HCV infection, n (%)^e^	93 (54%)	47 (48%)	0.35
Median ALT, U/L (IQR)	29 (22, 42)	29 (19, 44)	0.85
Median AST, U/L (IQR)	31.6 (24, 45)	28 (22.5, 43.5)	0.36
Median total cholesterol, mg/dL (IQR)	173 (151, 199)	176 (153, 200)	0.56
Median triglycerides, mg/dL (IQR)	120 (90, 198)	96 (73.5, 149.5)	0.002
Median high-density lipoprotein, mg/dL (IQR)	41 (33, 53)	44.5 (35.5, 57.5)	0.08
FIB-4, n (%)			< 0.001
< 1.45	75 (44%)	51 (53%)	
1.45–3.25	72 (42%)	28 (29%)	
> 3.25	21 (12%)	5 (5%)	
Missing	3 (2%)	13 (13%)	

Abbreviations: *ALT* alanine aminotransferase, *AST* aspartate aminotransferase, *ART* antiretroviral therapy, *FIB-4* fibrosis 4 score for liver fibrosis, *HCV* hepatitis C virus, *SAT* subcutaneous adipose tissue area, *VAT* visceral adipose tissue area

^a^Diabetes defined by International Classification of Diseases, Ninth Revision, diagnosis, anti-diabetic medication use, or random glucose > 200 mg/dL

^b^Hypertension defined by blood pressure ≥ 140/90 mmHg or use of anti-hypertensive medication

^c^Alcohol consumption determined by responses to the Alcohol Use Disorders Identification Test-Consumption questionnaire and alcohol dependence/abuse diagnoses

^d^ART use defined by use of at least three antiretrovirals from two different classes within 90 days of noncontrast abdominal CT scan

^e^HCV infection defined by positive HCV antibody or detectable HCV RNA

### Ethical considerations

Local Institutional Review Board or ethical review was obtained for all the three VACS clinical sites (Los Angeles Biomedical Research Institute, CA; University of Pittsburgh, PA; Vanderbilt University, TN), as well as for analytic and coordinating centers (University of Pittsburgh, PA; West Haven Veterans Affairs Medical Center, CT). Eligible participants were previously enrolled in the existing VACS and had signed informed consent granting permission for complete access to the VHA Health Information System [16]. After a full, face-to-face explanation of study purpose, risks and benefits, a new written informed consent for the additional data collection was obtained from each participant by local study site coordinators. Radiation history in the 12 months prior to this study enrollment was calculated from each eligible participant through listing of clinical radiographic procedures performed. Participants with estimated radiation exposure > 10 rems (Roentgen Equivalent Man) were excluded from the study to ensure radiation exposure would remain well below the dose limits set by FDA regulation 21CFR361.1 following completion of the proposed CT assessment.

## Results

Among 268 enrolled participants, 171 (63.8%) were HIV+ and 97 (36.2%) were uninfected. HIV+ participants were less commonly obese than uninfected participants, had lower median SAT area, but higher triglyceride levels (Table 1). Advanced hepatic fibrosis/cirrhosis by FIB-4 was more prevalent in HIV+ participants. Among those with HIV infection, the median CD4 cell count was 492 cells/mm^3^. For those on ART (156 [91%]), 141/156 (90%) had an undetectable HIV RNA.

The prevalence of hepatic steatosis (L/S < 1.0) was similar between HIV+ and uninfected participants (13 [7.6%; 95% CI, 3.6–11.6%] versus 8 [8.2%; 95% CI, 2.7–13.6%]; *p* = 0.85). After adjustment for race/ethnicity, alcohol use category, HCV infection, and FIB-4, in addition to features of the metabolic syndrome (i.e., obese BMI, hypertension, triglycerides, and diabetes), HIV was associated with a higher mean absolute liver attenuation, indicating less severe steatosis (Table 2). In multivariable linear regression models stratified by HIV status, advanced hepatic fibrosis/cirrhosis by FIB-4 remained the only factor associated with greater severity of steatosis among HIV+ and uninfected individuals, such that for FIB-4 > 3.25, the severity of steatosis was increased (i.e., decrease in HU units) by − 6.07 HU and − 18.22 HU, respectively.

**Table 2:** Mean difference in absolute liver attenuation (Hounsfield units [HU]) for variables of interest, overall and by HIV status^a^

Variable	Overall (*n* = 268)		HIV+ (n = 171)		HIV-(*n* = 97)	
	Mean difference (HU)		Mean difference (HU)		Mean difference (HU)	
	Crude (95% CI)	Adjusted (95% CI)	Crude (95% CI)	Adjusted (95% CI)	Crude (95% CI)	Adjusted (95% CI)
HIV	5.68 (2.93, 8.43)	5.97 (3.21, 8.73)	–	–	–	–
Race/ethnicity						
White	ref	ref	ref	ref	ref	ref
Black	− 1.41 (−5.43, 2.60)	0.06 (− 3.99, 4.11)	− 1.24 (− 6.06, 3.57)	-0.07 (−5.14, 5.28)	0.18 (− 6.85, 7.20)	-0.38 (−7.47, 6.71)
Hispanic	3.21 (−3.81,10.22)	2.43 (− 4.32, 9.17)	2.91 (−5.15, 10.98)	3.58 (− 4.62, 11.78)	2.30 (− 11.51, 16.10)	−2.03 (− 15.91, 11.85)
Other	0.56 (−10.07, 11.19)	2.16 (−7.88, 12.21)	0.42 (− 13.25, 14.09)	2.19 (−11.62, 16.00)	2.82 (−13.42, 19.06)	0.03 (− 15.27, 15.33)
Obese BMI	−4.59 (−7.79, −1.39)	−3.00 (−6.17, 0.18)	−2.31 (−6.86, 2.24)	−2.24 (− 7.06, 2.57)	− 4.80 (−9.18, − 0.43)	− 4.31 (−8.69, 0.07)
SAT area (cm^2^)	−0.02 (− 0.03, − 0.01)	–	− 0.02 (− 0.03, − 0.003)	–	− 0.02(− 0.03, − 0.007)	–
VAT area (cm^2^)	−0.03 (−0.04, − 0.01)	–	−0.02 (− 0.04, 0.008)	–	−0.03 (− 0.06, − 0.008)	–
Diabetes mellitus^b^	− 2.15 (− 5.58, 1.29)	−0.85 (−4.16, 2.47)	−2.93 (−7.24, 1.39)	−1.69 (− 6.18, 2.81)	− 0.72 (− 6.03, 4.59)	1.03 (− 4.02, 6.09)
Hypertension^c^	− 4.06 (−7.60, −0.53)	−1.08 (− 4.63, 2.46)	−4.21 (− 8.61, 0.18)	− 1.36 (− 6.12, 3.39)	−3.52 (− 9.08, 2.03)	−1.24 (− 7.11, 4.64)
Triglycerides	− 0.005 (−0.01, 0.005)	−0.006 (−0.02, 0.003)	− 0.008 (− 0.02, 0.003)	−0.008 (− 0.02, 0.004)	− 0.005 (−0.02, 0.01)	−0.001 (− 0.02, 0.01)
Alcohol use^d^						
Not current	ref	ref	ref	ref	ref	ref
Non-hazardous	4.67 (0.64, 8.69)	3.13 (−0.89, 7.14)	3.16 (−1.66, 7.98)	1.08 (−4.13, 6.28)	6.96 (0.04, 13.89)	5.51 (−1.27, 12.29)
Hazardous/at risk	7.64 (2.41,12.87)	5.76 (0.62, 10.91)	7.76 (0.97, 14.54)	4.32 (−2.88, 11.51)	8.99 (1.30, 16.68)	6.00 (−1.61, 13.62)
Abuse/dependence	2.18 (−0.93, 5.30)	1.95 (−1.07, 4.99)	1.58 (−2.38, 5.55)	0.99 (−3.14, 5.13)	4.65 (−0.11, 9.41)	2.68 (−2.15, 7.51)
Nadir CD4, cells/mm^3^	–	–	−0.003 (−0.01, 0.007)	–	–	–
Current CD4, cells/mm^3^	–	–	−0.0006 (−0.07, 0.006)	–	–	–
HIV viremia, log_10_	–	–	0.09 (−1.87, 2.06)	−0.12 (−2.13, 1.89)	–	–
HCV infection	−2.91 (− 5.61, −0.20)	−0.97 (−3.63, 2.24)	−4.83 (− 8.19, − 1.47)	−3.11 (−7.10, 0.88)	−0.43 (− 4.65, 3.79)	1.66 (−2.85, 6.16)
FIB-4						
< 1.45	ref	ref	ref	ref	ref	ref
1.45–3.25	−0.56 (−3.48, 2.35)	−0.71 (−3.63, 2.20)	−2.40 (−5.98, 1.19)	−1.09 (−4.95, 2.76)	0.95 (−3.55, 5.45)	0.24 (−4.56, 5.04)
> 3.25	−9.73 (−14.44, −5.01)	−9.43 (− 14.15, − 4.71)	−8.49 (− 13.93, − 3.04)	− 6.07 (− 11.96, − 0.18)	−17.64 (− 26.60, − 8.69)	−18.22 (− 27.64, − 8.81)

Abbreviations: *BMI* body mass index, *FIB-4* Fibrosis-4 Index for Liver Fibrosis, *HCV* hepatitis C virus, *SAT* subcutaneous adipose tissue area, *VAT* visceral adipose tissue area

^a^Absolute liver attenuation (in HU) has a linear, inverse association with severity of hepatic steatosis by pathology (i.e., higher HU indicates less hepatic steatosis)

^b^Diabetes defined by International Classification of Diseases, Ninth Revision, diagnosis, anti-diabetic medication use, or random glucose > 200 mg/dL

^c^Hypertension defined by blood pressure ≥ 140/90 mmHg or use of anti-hypertensive medication

^d^Alcohol use determined by responses to the Alcohol Use Disorders Identification Test-Consumption questionnaire and alcohol dependence/abuse diagnoses

In sensitivity analyses, higher BMI correlated with higher VAT (rho = 0.57) and SAT (rho = 0.79) area. Replacement of BMI with VAT or SAT in multivariable models did not significantly influence the magnitude of parameter estimates (Additional file 1: Tables S1 and S2). In exploratory analyses stratified by obese BMI and HIV status, hypertension and diabetes were associated with more severe steatosis among obese HIV+ persons, although not statistically significant. HCV infection was associated with more severe steatosis only among non-obese HIV+ individuals while advanced fibrosis was associated with more severe steatosis in uninfected individuals (Additional file 1: Table S3).

## Discussion

In this sample of HIV+ and uninfected US veterans, the prevalence of hepatic steatosis by noncontrast abdominal CT assessment was 7.8% overall, and similar in both groups. HIV was associated with significantly less severe hepatic steatosis after controlling for obesity, diabetes, hypertension, alcohol use, triglycerides, HCV infection and FIB-4. Advanced hepatic fibrosis/cirrhosis as measured by FIB-4 > 3.25 was associated with more severe steatosis in both HIV+ and uninfected individuals.

Prevalence estimates for hepatic steatosis among HIV+ persons have varied widely, ranging from 13 to 72% [5–13]. Cross-sectional studies evaluating the association between HIV and hepatic steatosis have produced varying results, with some reporting a positive association [6, 7, 12], negative association [5, 8, 11], or no association [10, 13]. These disparate findings may be a result of the different modalities employed to measure hepatic steatosis and the differing sensitivity and specificity of such methods. Liver biopsy is considered the diagnostic gold standard for hepatic steatosis; however, this approach can be subject to sampling bias, and observational studies restricted to only those referred for liver biopsy may enrich the study population for liver disease [28–30].

In our study of participants without pre-existing cardiovascular disease, we found that hepatic steatosis, as determined by noncontrast CT, was present in 7.8% overall and was not associated with HIV. The estimate of the prevalence of hepatic steatosis in our sample is much lower than the 17.6% estimate reported by Kanwal et al. in a much larger sample of 9,784,541 patients with at least one visit to the Veterans Health Administration between 2003 and 2011 [31]. Our study sample consisted predominantly of black men without cardiovascular disease. This might have led to our selecting patients who less frequently had obesity, diabetes, and hypertension and could have led to our finding lower estimates of the prevalence of hepatic steatosis among the HIV+ and uninfected participants compared to those from other studies [32–34]. However, our findings are similar to those reported from the Multicenter AIDS Cohort Study (MACS), which also used noncontrast abdominal CT to determine the presence of hepatic steatosis (defined by L/S < 1.0) and noted a lower prevalence in HIV+ (13%) than uninfected persons (19.2%) [11]. These results challenge the notion that HIV increases the risk of hepatic steatosis.

We also observed that HIV+ participants had less severe hepatic steatosis compared to uninfected individuals. This finding is consistent with a recent cross-sectional study that showed that HIV+ persons had quantitatively less hepatic steatosis, as measured by MRI liver fat fraction, than uninfected persons [15]. The lower severity of steatosis among HIV+ in these studies may be due to selection of HIV+ participants with more aggressive control of metabolic and cardiovascular comorbidities. Alternatively, HIV+ patients may have less caloric surplus than uninfected persons due to increased energy expenditure associated with HIV infection, which may prevent hepatic fat deposition [35, 36].

We found an association between advanced hepatic fibrosis (FIB-4 > 3.25) and increasing severity of hepatic steatosis, regardless of HIV status. This association was independent of HCV infection, alcohol use, diabetes, hypertension, triglycerides, or obesity. Excessive lipid deposition may potentiate hepatocyte injury through generation of reactive oxygen species during fatty acid oxidation, triggering a proinflammatory response [37]. Further oxidative stress and hepatocyte injury results in activation of hepatic stellate cells and subsequent collagen deposition, resulting in progressive fibrosis. Prior studies describing the histologic spectrum of non-alcoholic fatty liver disease have noted that hepatic steatosis can diminish during the late stages of cirrhosis [38] a contrast to our finding of increased steatosis severity in participants with FIB-4 > 3.25. This contrast may be due to the heterogeneity in severity of advanced fibrosis among out study participants as well as the small sample size of participants included. Further, while FIB-4 has been validated to identify advanced hepatic fibrosis/cirrhosis compared to liver biopsy with high positive predictive value, we cannot exclude the possibility that some individuals were misclassified by FIB-4 due to conditions which can transiently decrease the platelet count or increase liver aminotransferase levels [25]. Hepatic fibrosis would ideally be assessed with magnetic resonance elastography or shear wave elastography, which have the highest diagnostic accuracy for staging hepatic fibrosis among non-alcoholic fatty liver disease patients [39]; however, these modalities were not available to assess hepatic fibrosis during the time of this study (2008–2011).

In exploratory analyses among HIV+ patients stratified by obesity status, we found that hypertension and diabetes were associated with more severe steatosis among obese HIV+ persons, while HCV infection was associated with more severe steatosis among non-obese HIV+ individuals. These results suggest that obesity may modify the effect of some risk factors for hepatic steatosis in the setting of HIV infection. Due to the small numbers of obese HIV+ persons, these results should be interpreted with caution. Further study is warranted given the recent rise of obesity in HIV+ individuals [40–43].

Our study has several potential limitations. First, since cross-sectional studies evaluate exposure and disease status at the same point in time, this study design is limited in its ability to determine whether exposure preceded or resulted from disease. While noncontrast abdominal CT is a validated method for qualitative and quantitative assessment of hepatic steatosis, L/S ratio < 1.0 threshold has reduced sensitivity for the detection of mild steatosis and may underestimate the true prevalence [18, 20, 44–47]. Second, as previously noted, some individuals’ hepatic fibrosis stage might have been misclassified by FIB-4 due to conditions which decrease the platelet count or increase liver aminotransferases. Third, a large proportion (91%) of the HIV+ group was receiving ART, as is recommended by current management guidelines [24] and this limited our ability to examine associations between ART and hepatic steatosis. Future studies should evaluate whether ART or specific antiretrovirals contribute to the development of steatosis. Finally, our study sample consisted predominantly of men without cardiovascular disease. While the generalizability of our results may be limited to similar populations, our findings speak to the variable prevalence of hepatic steatosis and highlight the need to further understand how this disease process differs by HIV status.

## Conclusions

In conclusion, in this population of US veterans free of cardiovascular disease, the prevalence of hepatic steatosis was low and did not differ by HIV status. HIV infection was associated with less severe steatosis. Increasing severity of steatosis was associated with advanced hepatic fibrosis as measured by FIB-4 in both HIV+ and uninfected, suggesting more severe steatosis may potentiate fibrosis development. Future studies should compare the impact of hepatic steatosis on development of liver complications, such as cirrhosis and hepatocellular carcinoma, in HIV+ and uninfected persons.

## Additional file

Additional file 1: Supplemental Analyses. Multivariable linear regression models of mean difference in absolute liver attenuation adjusting for variables of interest, in addition to visceral adipose tissue (Table S1) or subcutaneous adipose tissue (Table S3), or stratified by obesity and HIV status (Table S3). (DOCX 21 kb)

## Abbreviations

ALT: Alanine aminotransferase
ART: Antiretroviral therapy
AST: Aspartate aminotransferase
BMI: Body mass index
CT: Computed tomography
FIB-4: Fibrosis-4 Index for Liver Fibrosis
HCV: Hepatitis C virus
HU: Hounsfield Units
L/S: Liver-to-spleen
SAT: Subcutaneous adipose tissue
VA: Veterans Health Administration
VACS: Veterans Aging Cohort Study
VAT: Visceral adipose tissue
