# Supplementary material for: Comparison of the prevalence, severity, and risk factors for hepatic steatosis in HIV-infected and uninfected people
Source: BMC Gastroenterol. 2019 Apr 15;19:52. doi: 10.1186/s12876-019-0969-1 (PMC6466708; doi:10.1186/s12876-019-0969-1)
Supplement: Supplementary file 1 — Supplemental Analyses. Multivariable linear regression models of mean difference in absolute liver attenuation adjusting for variables of interest, in addition to visceral adipose tissue (Table S1) or subcutaneous adipose tissue (Table S3), or stratified by obesity and HIV status (Table S3). (DOCX 21 kb) [file 12876_2019_969_MOESM1_ESM.docx]

| **Supplementary Table 1.** Mean difference in absolute liver attenuation (Hounsfield units [HU]) for variables of interest, replacing obese body mass index with visceral adipose tissue. | | |
| --- | --- | --- |
| **Variable** | **Overall**  **(n=268)** | |
|  | **Mean difference (HU)** | **Mean difference (HU)** |
|  | **Crude**  **(95% CI)** | **Adjusted**  **(95% CI)** |
| HIV | 5.68 (2.93, 8.43) | 6.07 (3.35, 8.79) |
| Race  White  Black  Hispanic  Other | ref  -1.41 (-5.43, 2.60)  3.21 (-3.81,10.22)  0.56 (-10.07, 11.19) | ref  -1.15 (-5.18, 2.88)  1.67 (-5.01, 8.36)  0.86 (-9.16, 10.88) |
| VAT (cm^2^) | -0.03 (-0.04, -0.01) | -0.02 (-0.05, -0.005) |
| Diabetes mellitus* | -2.15 (-5.58, 1.29) | -0.12 (-3.47, 3.23) |
| Hypertension^†^ | -4.06 (-7.60, -0.53) | -0.71 (-4.27, 2.85) |
| Triglycerides | -0.005 (-0.01, 0.005) | -0.005 (-0.01, 0.005) |
| Alcohol use^‡^  Not current  Non-hazardous  Hazardous/at risk  Abuse/dependence | ref  4.67 (0.64, 8.69)  7.64 (2.41,12.87)  2.18 (-0.93, 5.30) | ref  2.93 (-1.07, 6.93)  5.70 (0.58, 10.82)  1.38 (-1.70, 4.46) |
| HCV infection | -2.91 (-5.61, -0.20) | -1.64 (-4.62, 1.34) |
| FIB-4  <1.45  1.45-3.25  >3.25 | ref  -0.56 (-3.48, 2.35)  -9.73 (-14.44, -5.01) | ref  -0.66 (-3.56, 2.23)  -9.09 (-13.79,-4.40) |
| Abbreviations: FIB-4, fibrosis 4 score for liver fibrosis; HCV, hepatitis C virus; VAT, visceral adipose tissue area.  *Diabetes defined by ICD-9 diagnosis, anti-diabetic medication use, or random glucose >200 mg/dL.  ^†^Hypertension defined by blood pressure ≥140/90 mm Hg or use of anti-hypertensive medication.  ^‡^Alcohol use determined by responses to the Alcohol Use Disorders Identification Test-Consumption questionnaire and alcohol dependence/abuse diagnoses. | | |

| **Supplementary Table 2.** Mean difference in absolute liver attenuation (Hounsfield units [HU]) for variables of interest, replacing obese body mass index with subcutaneous adipose tissue. | | |
| --- | --- | --- |
| **Variable** | **Overall**  **(n=268)** | |
|  | **Mean difference (HU)** | **Mean difference (HU)** |
|  | **Crude**  **(95% CI)** | **Adjusted**  **(95% CI)** |
| HIV | 5.68 (2.93, 8.43) | 5.14 (2.38, 7.90) |
| Race  White  Black  Hispanic  Other | ref  -1.41 (-5.43, 2.60)  3.21 (-3.81,10.22)  0.56 (-10.07, 11.19) | ref  0.40 (-3.57, 4.37)  3.04 (-3.59, 9.68)  3.78 (-6.14, 13.70) |
| SAT (cm^2^) | -0.02 (-0.03, -0.01) | -0.016 (-0.02,-0.007) |
| Diabetes mellitus* | -2.15 (-5.58, 1.29) | -0.88 (-4.13, 2.38) |
| Hypertension^†^ | -4.06 (-7.60, -0.53) | -0.66 (-4.14, 2.83) |
| Triglycerides | -0.005 (-0.01, 0.005) | -0.006 (-0.02, 0.004) |
| Alcohol use^‡^  Not current  Non-hazardous  Hazardous/at risk  Abuse/dependence | ref  4.67 (0.64, 8.69)  7.64 (2.41,12.87)  2.18 (-0.93, 5.30) | ref  2.72 (-1.23, 6.68)  5.72 (0.67, 10.77)  1.62 (-1.36, 4.60) |
| HCV infection | -2.91 (-5.61, -0.20) | -1.16 (-4.04, 1.72) |
| FIB-4  <1.45  1.45-3.25  >3.25 | ref  -0.56 (-3.48, 2.35)  -9.73 (-14.44, -5.01) | ref  -0.82 (-3.68, 2.04)  -9.26 (-13.95,-4.56) |
| Abbreviations: FIB-4, fibrosis 4 score for liver fibrosis; HCV, hepatitis C virus; VAT, visceral adipose tissue area.  *Diabetes defined by ICD-9 diagnosis, anti-diabetic medication use, or random glucose >200 mg/dL.  ^†^Hypertension defined by blood pressure ≥140/90 mm Hg or use of anti-hypertensive medication.  ^‡^Alcohol use determined by responses to the Alcohol Use Disorders Identification Test-Consumption questionnaire and alcohol dependence/abuse diagnoses. | | |

| **Supplementary Table 3**. Adjusted mean difference in absolute liver attenuation (Hounsfield units [HU]) for variables of interest, by obese body mass index and HIV status. | | | | |
| --- | --- | --- | --- | --- |
| **Variable** | **BMI ≥ 30** | | **BMI <30** | |
|  | **HIV+**  **(n=29)*** | **HIV-**  **(n=25)*** | **HIV+**  **(n=141)*** | **HIV-**  **(n=59)*** |
|  | **Mean difference (HU)**  **(95% CI)** | **Mean difference (HU)**  **(95% CI)** | **Mean difference (HU)**  **(95% CI)** | **Mean difference (HU)**  **(95% CI)** |
| Race  White  Black  Hispanic  Other | ref  -8.3 (-28.0, 11.4)  -7.9 (-31.8, 16.0)  -- | ref  11.9 (-12.0, 35.8)  --  11.9 (-19.8, 43.7) | ref  2.2 (-3.4, 7.8)  7.0 (-2.3, 16.4)  3.4 (-10.5, 17.4) | ref  -6.2 (-14.6, 2.3)  -7.5 (-22.6, 7.6)  -5.7 (-26.5, 15.2) |
| Diabetes mellitus^†^ | -6.3 (-18.8, 6.1) | 1.1 (-10.2, 12.4) | -0.9 (-6.0, 4.2) | -1.7 (-7.9, 4.5) |
| Hypertension^‡^ | -5.8 (-25.6, 13.9) | 5.8 (-10.8, 22.4) | -0.006 (-5.1, 5.1) | -1.2 (-8.1, 5.7) |
| Triglycerides | 0.004 (-0.02, 0.03) | -0.009 (-0.06, 0.04) | -0.02 (-0.04, -0.003) | -0.002 (-0.02, 0.02) |
| Alcohol use^§^  Not current  Non-hazardous  Hazardous/at risk  Abuse/dependence | ref  10.6 (-6.5, 27.7)  10.4 (-6.7, 27.6)  1.7 (-13.6, 17.1) | ref  6.0 (-12.9, 24.8)  15.5 (-9.4, 40.3)  5.0 (-4.8, 14.7) | ref  -1.3 (-7.2, 4.6)  3.6 (-4.9, 12.1)  -0.4 (-4.9, 4.0) | ref  1.1 (-7.3, 9.5)  0.8 (-8.9, 10.6)  0.4 (-5.8. 6.5) |
| Current CD4, cells/mm^3^ | -0.004 (-0.02, 0.01) | -- | -0.003 (-0.01, 0.004) | -- |
| HIV viremia, log_10_ | -1.6 (-6.2, 3.1) | -- | -0.3 (-2.9, 2.3) | -- |
| HCV infection | 8.5 (-5.4, 22.4) | -1.5 (-13.0, 9.9) | -5.4 (-9.7, -1.0) | 2.2 (-3.3, 7.7) |
| FIB-4  <1.45  1.45-3.25  >3.25 | ref  -2.4 (-15.3, 10.4)  -10.6 (-27.7, 6.6) | ref  3.7 (-9.9, 12.3)  -- | ref  -1.3 (-5.4, 2.9)  -4.8 (-11.3, 1.7) | ref  2.1 (-3.3, 7.4)  -21.9 (-30.8, -13.9) |
| Abbreviations: FIB-4, fibrosis 4 score for liver fibrosis; HCV, hepatitis C virus.  *Missing observations for following strata: 7 (22%) of obese HIV-, 1 (0.7%) non-obese HIV+, 6 (9%) non-obese HIV-.  ^†^Diabetes defined by ICD-9 diagnosis, anti-diabetic medication use, or random glucose >200 mg/dL.  ^‡^Hypertension defined by blood pressure ≥140/90 mm Hg or use of anti-hypertensive medication.  ^§^Alcohol use determined by responses to the Alcohol Use Disorders Identification Test-Consumption questionnaire and alcohol dependence/abuse diagnoses. | | | | |
